# FlowUTI: An interactive web-application for optimizing the use of flow cytometry as a screening tool in urinary tract infections

**DOI:** 10.1371/journal.pone.0277340

**Published:** 2022-11-08

**Authors:** Guillermo Martín-Gutiérrez, Carlos Martín-Pérez, Héctor Toledo, Emilio Sánchez-Cantalejo, José Antonio Lepe

**Affiliations:** 1 Clinical Unit of Infectious Diseases, Microbiology and Parasitology, University Hospital Virgen del Rocío, Seville, Spain; 2 Instituto de Biomedicina de Sevilla (IBiS), Sevilla, Spain; 3 Centro de Investigación Biomédica en Red de Enfermedades Infecciosas, Madrid, Spain; 4 Retired, Spain; University of Jeddah, SAUDI ARABIA

## Abstract

Due to the high prevalence of patients attending with urinary tract infection (UTI) symptoms, the use of flow-cytometry as a rapid screening tool to avoid unnecessary cultures is becoming a widely used system in clinical practice. However, the recommended cut-points applied in flow-cytometry systems differ substantially among authors, making it difficult to obtain reliable conclusions. Here, we present FlowUTI, a shiny web-application created to establish optimal cut-off values in flow-cytometry for different UTI markers, such as bacterial or leukocyte counts, in urine from patients with UTI symptoms. This application provides a user-friendly graphical interface to perform robust statistical analysis without a specific training. Two datasets are analyzed in this manuscript: one composed of 204 urine samples from neonates and infants (≤3 months old) attended in the emergency department with suspected UTI; and the second dataset including 1174 urines samples from an elderly population attended at the primary care level. The source code is available on GitHub (https://github.com/GuillermoMG-HUVR/Microbiology-applications/tree/FlowUTI/FlowUTI). The web application can be executed locally from the R console. Alternatively, it can be freely accessed at https://covidiario.shinyapps.io/flowuti/. FlowUTI provides an easy-to-use environment for evaluating the efficiency of the urinary screening process with flow-cytometry, reducing the computational burden associated with this kind of analysis.

## Introduction

Urinary tract infections (UTIs) are among the most common bacterial infections in humans, representing the second most frequent community-acquired infection in women [1, 2]. That being so, it is no wonder that urine specimens are one of the most received samples in clinical microbiology laboratories [3]. Urine culture is the gold-standard method for UTI to detect the causative agent and initiate an appropriate antibiotic treatment. However, most of the samples have a negative result (over 60%) [4–6], consuming time and resources in their processing, and thus increasing workload and costs.

Several screening tests are used in clinical microbiology laboratories to rule out negative urine samples. In this sense, flow cytometry has become one of the most used screening tools, allowing to inform negative results earlier, reducing time, costs and unnecessary empirical antibiotic treatments [7–9]. Nevertheless, although the usefulness of flow-cytometry as a screening tool has been well established for patients with UTI symptoms, bacteria and leukocyte cut-off values applied vary significantly across studies [10]. This issue can be explained by two main facts: i) the basic measures to validate the diagnostic tests are expected to vary strongly depending on disease prevalence [11], and UTI prevalence varies greatly with age, gender and underlying diseases [2, 12, 13]; ii) Additionally, the interpretation of the quantitative results obtained depends on the threshold applied, which also varies according to the clinical characteristics of the patients [14]. Therefore, due to the heterogeneity of the patients analyzed in the different studies, it is difficult to compare the cut-off values applied or to derive reliable conclusions. Consequently, the best choice for microbiology laboratories is to set their own cut-off values according to patients attended in their health centers, in order to improve the reliability of the screening.

Well-designed interactive tools empower users to integrate information and discover new associations without requiring programming knowledge or data-analytic skills. Considering the powerful functions in R [15], a practical open language for statistical and graphical exploration of data set, and the versatility of Shiny [16], a free web application that does not require knowledge of any programming language, we developed FlowUTI, the first openly available shiny web-application created to determine optimal cut-off values for different UTI markers from flow cytometry systems. In addition, with the aim of evaluating FlowUTI, two datasets obtained with two different flow cytometry systems are analyzed in this manuscript.

## Methods

### FlowUTI development

The source code of the web application is written in the R language [15], developed by using the function “epi.test” of the R-package epiR [17] and the R-package pROC [18]. We used a specific code to compute the most consistent diagnostic parameters used in clinical studies. Data manipulation and data analysis were executed using R-scripts, which rely on CRAN packages including: ggplot2, dplyr, xtable, shinythemes, knitr and rmarkdown. A shiny web application [16] was created with the aim of providing an accessible tool for non-R users. We used the web application server Shiny to create a robust graphical user interface, which allows an interactive manipulation and interaction with the application.

### Workflow

**Fig 1:**
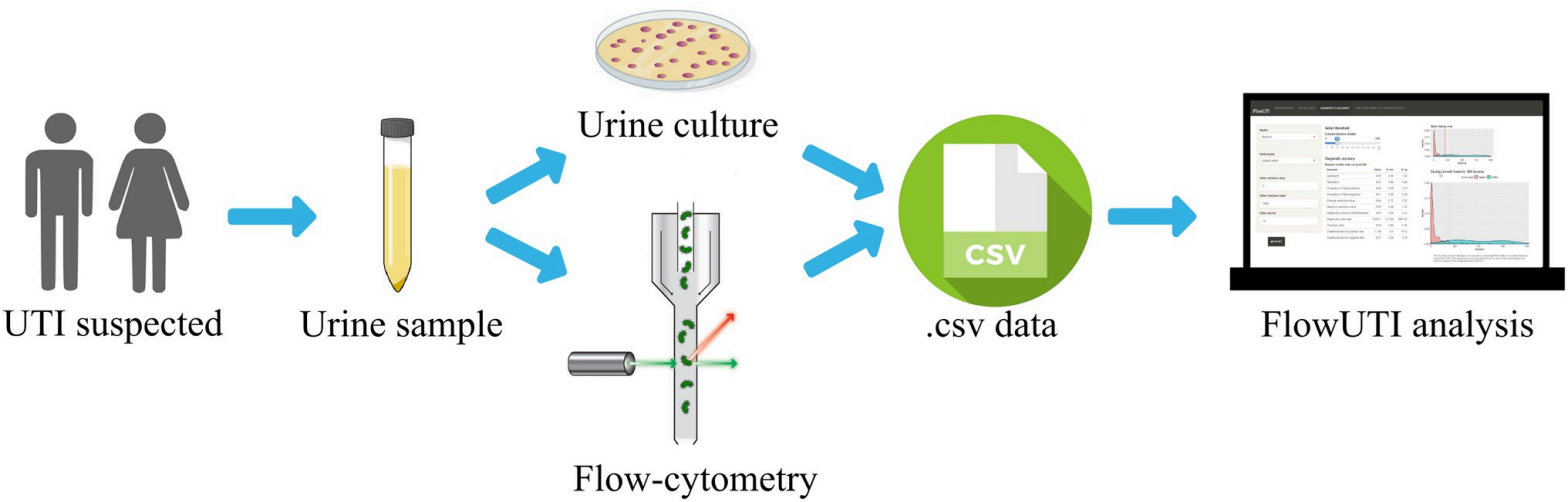
FlowUTI workflow. Flow cytometry figure was adapted from www.cytognos.com, and csv figure from www.shareicon.net.

Fig 1 shows the workflow graph. First, urines from patients with a suspected UTI are cultured according to national or international guidelines for the diagnosis of UTIs [19, 20]. Additionally, urines are analyzed by flow-cytometry according to the manufacturer’s instructions. Once urines have been analyzed, the results are transferred to a spreadsheet software. The minimum data set required to run the application comprises the culture result, that must be a dichotomous variable (that hold precisely two distinct values, such as “Positive” and “Negative” results), and the variable of interest obtained by flow cytometry, such us bacteria count. The data set can be supplemented with additional variables (leukocyte, epithelial cells, yeasts, etc.). Finally, each spreadsheet is saved as its own Comma Separated Value (.csv) file. These .csv files are easily obtained from Excel or R software.

### Using the application

FlowUTI is able to work with data from any flow-cytometry analyzer, regardless of the brand or model. First, in the *Upload data* section, the users will upload to FlowUTI the .csv file. Additionally, in the *Upload data* section, two demo datasets can be uploaded as examples of the data format needed for running the model, where the users can learn and be trained to work with the application before introducing their own data. The information about the patients’ characteristics included in these datasets is described further on. Once the document is uploaded, the user can preview the dataset.

**Fig 2:**
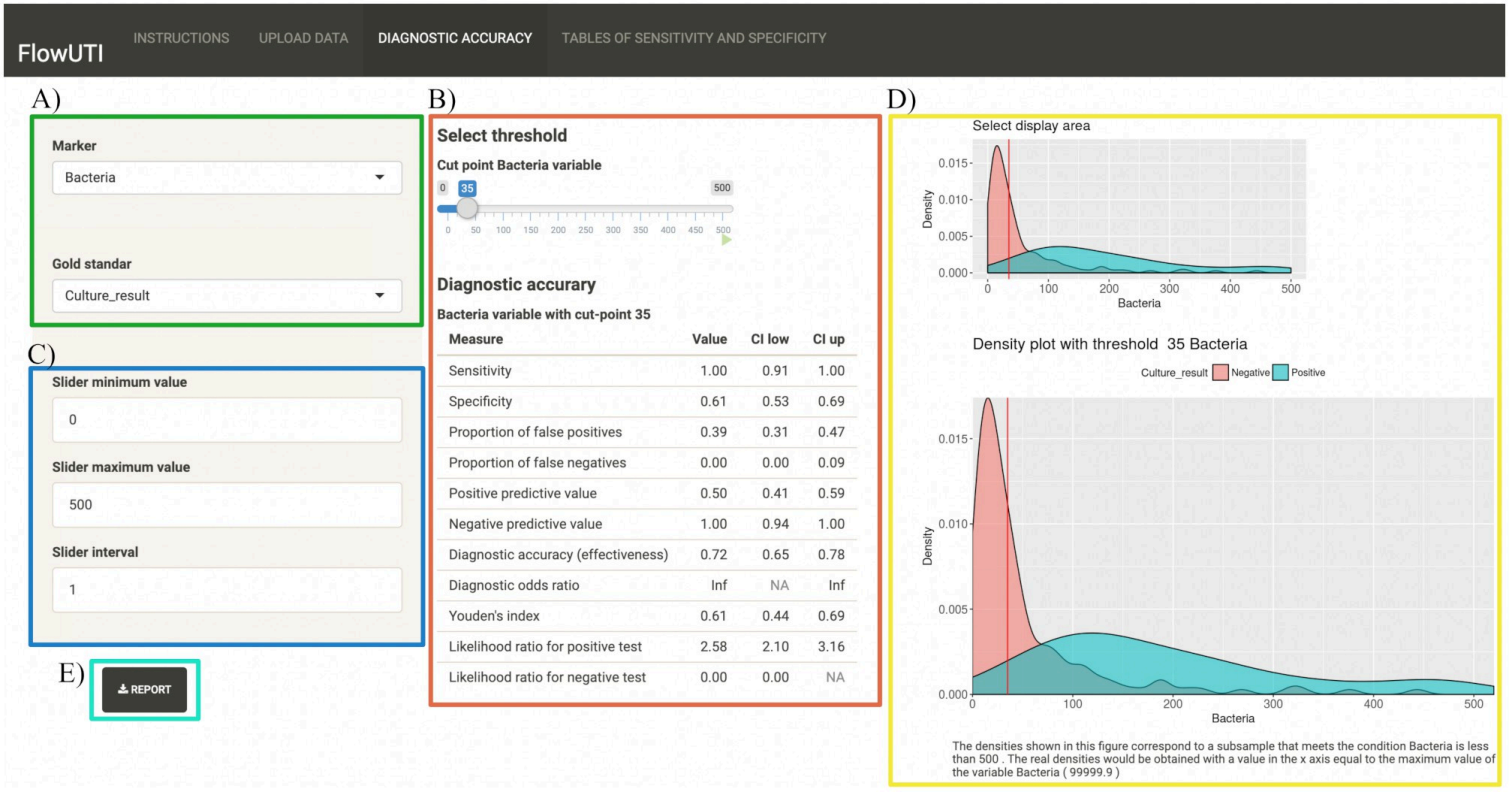
Screenshot of the FlowUTI diagnostic accuracy browser, showing the results obtained for bacterial counts corresponding to the neonate demo dataset.

In the section *Diagnostic accuracy*, the user can explore the diagnostic parameters obtained according to the selected cut-off value (Fig 2). Firstly, the user selects the independent variable of concern (bacteria or leukocyte) and the dependent variable (culture result, the gold standard) (Fig 2A). After that, the cut-off values can be selected by clicking on the slider bar. Immediately, the application will run the computations and will display a table with all diagnostic parameters obtained according to the selected cut-off (Fig 2B). The lower and higher values for bacteria or leukocytes can be chosen to optimize the selection of the cut-off value (Fig 2C). In addition, the application generates a density distribution graph (Fig 2D), showing the positive and negative cultures distribution according to bacteria or leukocyte counts. In this interactive chart the users can zoom in and out, which will help visually to consider the best cut-off value. In this section, the users can also generate a summary document in .pdf format by clicking in the “Report” button (Fig 2E), gathering all the results according to the selected cut-off value, depending on the user’s choice.

Finally, in the section *Tables of sensitivity and specificity*, the discrimination power of bacteria and leukocyte counts are assessed by Receiver Operating Characteristic (ROC) curve analysis. As was performed in the last section, the user selects the independent and dependent variables of interest, and then the sensitivity, specificity, and the area under the curve (AUC), which is the measure of the ability of a classifier to distinguish between classes, used as a summary of the ROC curve (the higher the AUC, the better the performance of the model at distinguishing between the positive and negative classes), are immediately calculated using bootstrapping (n = 1000). Additionally, placed at the right side, the sensitivity-specificity and ROC curves are plotted.

### Web application

We host the web application on the Shiny server at the web address https://covidiario.shinyapps.io/flowuti/. The user’s database is held temporarily in the application and will be discarded when the session is terminated. Each data upload is only viewable by the user during that single browser session. We will ensure that the FlowUTI app is online for five years following the publication date, with a monthly usage limit of 25 hours. The source code of FlowUTI is available on GitHub (https://github.com/GuillermoMG-HUVR/Microbiology-applications/tree/FlowUTI/FlowUTI), which can be downloaded into any computer to be used offline without limitations by using the R software.

### Datasets

With the aim of evaluating FlowUTI, two datasets are analyzed in this manuscript. One of them (Neonate .csv) is composed of 204 urine samples from neonates and infants (≤ 3 months old) attended in the emergency department of the University Hospital Virgen del Rocío with suspected UTI during the years 2013–2015. All urine samples were obtained by bladder catheterization and held at 4°C until processed. All samples were processed within 2 hours after collection for culture and flow-cytometry analysis (UF-1000i, Sysmex). Counts above ≥10^2^ colony-forming units (CFU)/ml were considered as positive cultures [19]. Regarding the second dataset (Elderly .csv), 1174 urine samples were included from an elderly population (≥65 years old) with UTI symptoms attended at the Primary Care Units of Virgen del Rocío University Hospital from January to December 2021. All samples were processed within 4–6 hours after collection for culture and flow-cytometry analysis (UF-5000, Sysmex). Counts above ≥10^5^ CFU/ml were considered as positive cultures [21]. Both datasets are available on GitHub (https://github.com/GuillermoMG-HUVR/Microbiology-applications/tree/FlowUTI/FlowUTI/Demos).

## Results

### Examples of use

Below, we describe the use of FlowUTI for the screening of UTIs at the extreme-age groups: neonates and elderly. It is important to remark that the objective of this manuscript is not to determine a general cut-off value for these two groups of patients, but to highlight the importance of determining the cut-off values according to the patients analyzed. Therefore, the cut-offs evaluated by this study may not be useful in other centers.

#### Neonate dataset

Once the Neonate .csv file is uploaded, we can observe a total of 204 records and two independent variables: bacteria and leukocytes. Table 1 summarizes the results obtained from the section *Diagnostic accuracy*. As can be observed, by selecting a cut-off value of 35 bacteria, a sensitivity of 100% is obtained, without false negative results, and with specificity and false positivity rates of 61% and 39%, respectively. Moreover, with this cut-off value we could avoid the culture of 44.11% of the urine samples. The higher the bacterial count selected as threshold, the higher the false negative rates. In the section *Tables of sensitivity and specificity*, an AUC of 0.948 was obtained, which means that bacterial count has an excellent discrimination capability for UTIs. On the other hand, leukocyte count appears not be as effective as the bacterial count as a screening of UTIs in this dataset, with an AUC of 0.808.

**Table 1:** Bacteria and leukocyte count in urine specimens from neonates.

Bacteria	% (CI)						
	SE	SP	FP	FN	PPV	NPV	EF
35	100 (91–100)	61 (53–69)	39 (31–47)	0 (0–9)	50 (41–59)	100 (94–100)	72 (65–78)
40	98 (91–100)	65 (56–72)	35 (28–44)	2 (0–9)	52(42–62)	99(94–100)	74 (67–80)
45	98 (91–100)	67 (59–75)	33 (25–41)	2 (0–9)	54 (44–64)	99(95–100)	76(69–82)
50	96 (88–100)	73 (65–80)	27 (20–35)	4 (0–12)	58 (47–68)	98 (94–100)	79 (73–85)
60	95 (85–99)	75 (67–82)	25 (18–33)	5 (1–15)	59 (48–69)	97 (92–99)	80 (74–86)
70	95 (85–99)	78 (70–84)	22 (16–30)	5 (1–15)	62 (51–72)	97 (93–99)	82 (76–87)
100	88 (76–95)	85 (78–90)	15 (10–22)	12 (5–24)	69 (57–80)	95 (89–98)	86 (80–90)
**Leukocyte**							
1	100 (91–100)	3 (1–8)	97 (92–99)	0 (0–9)	29 (23–35)	100 (36–100)	30 (24–37)
5	93 (83–98)	24 (17–32)	76 (69–83)	7 (2–17)	32 (25–40)	90 (76–97)	43 (36–50)
10	90 (79–96)	46 (37–54)	54 (46–63)	10 (4–21)	39 (30–48)	92 (83–97)	58 (51–65)
15	86 (74–94)	59 (51–67)	41 (33–49)	14 (6–26)	45 (35–55)	92 (84–96)	67 (60–73)
20	81 (68–90)	67 (58–74)	33 (26–42)	19 (10–32)	48 (38–59)	90 (83–95)	71 (64–77)
30	70 (57–82)	73 (66–80)	27 (20–34)	30 (18–43)	51 (39–62)	86 (79–92)	72 (66–79)

SE: Sensitivity; SP: Specificity; FP: False positive; FN: False negative, PPV: Positive Predictive Value; NPV: Negative Predictive Value, EF: Effectiveness; CI: Confidence Interval 95%.

#### Elderly dataset

In total, 1174 urine samples were included in the Elderly dataset (153 contaminated urines were discarded). We can observe that, with a bacteria cut-off value of 200 bacteria, a sensitivity of 95% is obtained, with a 5% of false negative results, a low value for sensitivity (56%) and a proportion of false positive of 44%. With these results, we could avoid the culture of 400 urine samples (34,07%). Again, the higher the bacterial count selected, the higher the false negative rates (Table 2). The leukocyte count presents low specificity values. Finally, the AUCs for bacterial and leukocytes counts are 0.924 and 0.79, respectively.

**Table 2:** Bacterial and leukocyte counts in urine specimens from elderly patients.

Bacteria	% (CI)						
	SE	SP	FP	FN	PPV	NPV	EF
100	97 (95–98)	42 (38–46)	58 (54–62)	3 (2–5)	55 (52–58)	95 (92–97)	65 (62–68)
150	96 (94–97)	50 (46–54)	50 (46–54)	4 (3–6)	58 (55–62)	94 (92–97)	69 (67–72)
200	95 (93–97)	56 (52–59)	44 (41–48)	5 (3–7)	61 (58–65)	94 (92–96)	72 (70–75)
300	94 (92–96)	64 (60–68)	36 (32–40)	6 (4–9)	66 (62–69)	94 (91–96)	77 (74–79)
400	93 (90–95)	69 (65–72)	31 (28–35)	7 (5–10)	68 (65–72)	93 (90–95)	79 (76–81)
**Leukocyte**							
5	99 (97–100)	10 (8–13)	90 (87–92)	1 (0–3)	45 (42–48)	92 (83–97)	48 (45–51)
10	97 (95–98)	16 (14–19)	84 (81–86)	3 (2–5)	46 (43–49)	87 (80–93)	50 (47–53)
20	94 (91–95)	27 (24–31)	73 (69–76)	7 (4–9)	48 (45–52)	85 (80–90)	55 (53–58)
30	91 (88–93)	36 (33–40)	64 (60–67)	9 (7–12)	51 (48–54)	84 (80–88)	59 (56–62)
50	88 (85–91)	47 (43–51)	53 (49–56)	12 (9–15)	55 (51–58)	84 (80–88)	65 (62–67)
100	83 (79–86)	59 (55–62)	41 (38–45)	17 (14–21)	59 (56–63)	82 (79–85)	69 (66–71)

SE: Sensitivity; SP: Specificity; FP: False positive; FN: False negative, PPV: Positive Predictive Value; NPV: Negative Predictive Value, EF: Effectiveness; CI: Confidence Interval 95%.

## Discussion

In this manuscript we present FlowUTI, a new interactive shiny web-application addressed to physicians and researchers interested in implementing and optimizing the use of flow-cytometry analyzers as a UTI screening method. FlowUTI is a free tool that provides a user-friendly graphical interface to perform robust statistical analysis without a specific training, maximizing the efficiency of the urinary screening process.

The selection of appropriate cut-off values of a screening method is crucial to avoid erroneous diagnosis in clinical practice. Usually, the choice of cut-off values for bacteria or leukocytes are in accordance with the criterion formerly established by prior studies. However, there is a substantial heterogeneity of cut-off values applied among the studies, making it difficult to select one in particular. This issue was addressed in the meta-analysis performed by Shang and colleagues [22], where a dramatic heterogeneity (*I*^2^ > 90%) on sensitivity, specificity, likelihood ratios and diagnostic odds ratios for both bacteria and leukocytes counts was observed. Another problem to be solved is the statistical methodology required to establish a general cut-off value by means of a meta-analysis, which is complex and cumbersome. Only recently a new R-package has been developed for this purpose [23], but an optimal threshold across all urine flow-cytometry studies has not been achieved yet.

The selection of the best cut-off value depends on the prevalence of UTIs in the population, the diagnostic threshold selected, and the type of flow-cytometer used. Furthermore, the clinical relevance of the UTI is also a key factor. In this way, it’s only possible to determine the most appropriate cut-off value carefully considering the clinical cost of false-negative and false-positive results. UTIs in neonates and infants are associated with significant morbidity and long-term medical consequences [24, 25], and because antibiotic treatments have a profound impact on the gut microbiota [26], it’s crucial to promptly and accurately identify those patients who do not have the UTI. For this reason, high values of sensitivity and negative predictive values (NPV) are needed to limit false negative findings, even if the number of false-positive findings is high, resulting in additional cultures. Conversely, the utility of flow-cytometry in the elderly is challenging because of the high prevalence of bacteriuria and pyuria that may not be clinically relevant [27, 28]. For this reason, it’s important to perform a clinical evaluation about what is an acceptable number of false positive and false negative results. Moreover, the significant number of contaminated specimens in these patients [29] may cause erroneous interpretation of urine cultures, masking true infections, or leading to unnecessary treatments until diagnosis [30].

FlowUTI is an open interactive application, which allows users to include different variables related with the UTI screening. For instance, the presence of squamous epithelial cells in urine during the sample collection procedure has been related with contaminated culture results [31]. Hence, users can include the epithelial cell counts and the “contaminated” result in the database, and then study the discrimination power of this variable and its utility to reduce the number of false positive results. Additionally, higher yeast-like cell counts by UF-5000 have been related with the presence of *Candida* spp. in urine samples [32], so it could be also interesting to evaluate this marker in selected patients [33].

In conclusion, we expect that FlowUTI might be a useful tool for physicians and researchers, allowing them to interactively intervene on the choice of the best cut-off values, making it easier to analyze the data obtained from flow-cytometry and reducing the computational burden associated with this kind of analysis, without losing statistical reliability. We encourage clinical microbiology laboratories to establish their own cut-off values according to the patients attended. By doing so, they could decrease turnaround time of analysis and improving clinical decisions.
